# Association between Diagnostic History and Cancer Incidence within 5 Years: A Real-world Observational Analysis

**DOI:** 10.1158/2767-9764.CRC-26-0163

**Published:** 2026-05-11

**Authors:** Md Ashad Alam, Grace Williams, Muhammad G. Kibriya, Marc Matrana, Nicholas Duesbery, Edward Trapido, Daniel Fort

**Affiliations:** 1 Ochsner Center for Outcomes Research, Ochsner Research, Ochsner Clinic Foundation, New Orleans, Louisiana.; 2 Louisiana Cancer Research Center, New Orleans, Louisiana.; 3 Public Health Sciences, Biological Sciences Division, University of Chicago, Chicago, Illinois.; 4 Ochsner MD Anderson Cancer Center, Ochsner Health, New Orleans, Louisiana.; 5 Louisiana State University School of Public Health, New Orleans, Louisiana.

## Abstract

Cancer is associated with many preexisting health conditions (PHC); however, accurately quantifying these links remains challenging. Although some studies have examined these associations, large-scale analyses using diverse electronic health record (EHR) data remain limited and lack the ability to evaluate cancer risk when patients are stratified by interpersonal differences. Using a real-world EHR dataset from a large Louisiana health system comprising 8,283,236 records from 1,460,738 patients (2013–2022), we evaluated associations between PHCs and subsequent cancer diagnoses within a fixed 5-year risk window. We applied epidemiologic, statistical, and artificial intelligence methods to the full dataset and to subgroups stratified by gender, race, and area deprivation index (ADI) for overall cancer and 20 cancer types. We identified 9 chapters of International Classification of Diseases, Tenth Revision, including chapter 4 (metabolic) and chapter 14 (genitourinary), with 221 PHCs linked to increased cancer risk (relative risk >1, 95% confidence interval excluding 1, and Benjamini–Hochberg false discovery rate–adjusted *P* < 0.05). Key PHCs include systemic sclerosis, blood type, benign mammary dysplasia, immune mechanism disorders, disturbances of smell, lipoprotein metabolism disorders, human immunodeficiency virus disease, vitamin D deficiency, and diabetes. Chapter 12 (skin diseases) and chapter 9 (circulatory diseases) showed strong associations with 10 and 13 cancer types, respectively. Age-, gender-, race-, and ADI-specific high-risk PHCs were also identified. However, these findings should be interpreted carefully as ADI may not fully capture individual-level socioeconomic or environmental exposures, and the lack of tobacco data may introduce residual confounding.

**Significance:**

This framework offers a systematic approach to linking PHCs with cancer risk, providing valuable insights for cancer prediction, management, and prevention across diverse patient populations. This approach reveals prediagnostic disease patterns and demographic heterogeneity in cancer incidence.

## Introduction

Cancer, a major global public health concern, is projected to surpass 35 million cases annually by 2050, marking a 77% increase from the estimated 20 million cases in 2022 (1–3). It ranks as the second leading cause of death in the United States. Many diseases are associated with cancer, and patients with cancer often have varied histories of hospital visits before receiving their initial diagnosis (4). Accurately quantifying specific preexisting health conditions (PHC) associated with cancer, as well as taking steps to predict the development of cancer, within large cohorts is a major obstacle in cancer research (5, 6). As the risk of cancer varies by gender and race and increases with age, patient demographics also play a key component in understanding who is most at risk of developing cancer (7–12). For instance, data collected through the Louisiana Tumor Registry demonstrates that the incidence of different types of cancer varies across the state of Louisiana by gender, race, ethnicity, and geographic location (13–18). The aim of this project is to investigate potential complex associations underlying these differences more deeply.

In recent years, researchers have introduced innovative methods for analyzing real-world, imaging genetics, imaging multiomics, and multiomics data, applying them to the study of overall cancer and specific cancer types (19–22). Moving beyond traditional case–control or cross-sectional studies, which have predominantly been employed to identify interaction effects in data modalities, this article addresses the increasing demand for characterizing PHC variants across the comprehensive, real-world data from electronic health records (EHR; refs. 23–29).

Several studies have examined the impact of PHCs on cancer diagnosis and management (30–32). However, comprehensive analyses of overall and 20 specific cancer types using large-scale real-world EHR data are limited (33). Research that includes stratified analyses by age, gender, race, ethnicity, and area deprivation index (ADI) in diverse US populations is also lacking. The objective of this study was to evaluate associations between PHCs and incident cancer within a fixed 5-year risk window and to examine how these associations vary by gender, race, and ADI. We applied a structured analytic framework using large-scale EHR data to identify PHCs significantly associated with overall cancer and 20 specific cancer types based on subheadings of International Classification of Diseases, Tenth Revision, Clinical Modification (ICD-10-CM). Analyses were conducted in the overall cohort and further stratified by age, gender, race, and ADI to assess subgroup differences. This framework enables systematic identification of PHCs associated with subsequent cancer diagnosis and may inform future risk stratification, screening strategies, and hypothesis generation in cancer research. However, ADI may not fully capture individual-level socioeconomic or environmental exposures, and the lack of tobacco data may introduce residual confounding.

## Materials and Methods

**Figure 1.**
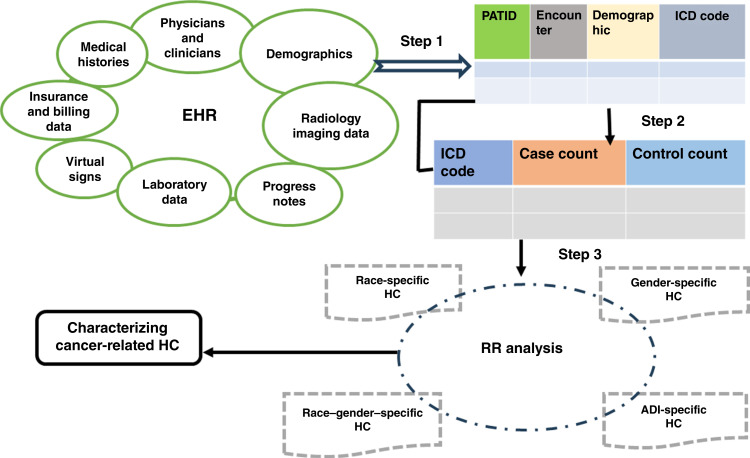
Three-step analytic framework for identifying cancer-related pre-HCs from EHR data. Step 1: structured extraction of patient encounters, demographics, and ICD codes from multimodal EHR components. Step 2: construction of case–control matrices with ICD-specific counts. Step 3: RR analysis performed overall and within demographic subgroups (race-specific, gender-specific, race–gender–specific, and ADI-specific) to characterize cancer-related HCs.

We utilized an extensive EHR dataset comprising real-world diagnosis records from Ochsner Health’s EHR over a 10-year span (2013–2022) to identify PHCs associated with a subsequent cancer diagnosis within 5 years of the initial PHC. This study was approved by an institutional review board. Supplementary Figure S1 displays a CONSORT diagram for the present study. The dataset consisted of 8,283,236 records from 1,460,738 patients 18 to 85 years of age, of whom 336,849 had complete information (Supplementary Fig. S1). An additional 877,258 patients were excluded as their first encounter with Ochsner Health was a cancer diagnosis and 30 days prior to all diagnoses. Cases and control subjects were defined by cancer diagnosis status; potential bias was mitigated through stratification by age, gender, race, and ADI (the residential address recorded at the first visit), and no formal power calculation was performed because all eligible patients in the cohort were included. For the case group of 291,638 patients with cancer, we included medical diagnosis codes from the 5 years immediately preceding the first cancer diagnosis but excluded codes from 30 days prior. The control group consisted of 45,211 patients without cancer. The control sample size was smaller because of the restriction to individuals without a baseline cancer diagnosis and with index dates between 2013 and 2018, ensuring at least 5 years of cancer-free follow-up. Across both groups, there were 10,624 common ICD-10-CM medical diagnosis codes (1,372 truncated), covering 20 ICD-10-CM chapters (Supplementary Table S1; ref. 34). For a comprehensive understanding of cancer-related PHCs, we used various statistical tests, including mean tests, *χ*^2^ tests, relative risk (RR) analysis (standard and binomial), prevalence analysis, Cohen d effect size, and gender/race difference tests (see Supplementary Materials and Methods). Figure 1 illustrates the detailed framework used in this study. We calculated RR for cancer diagnoses within 5 years by comparing patients with cancer (cases) with those without a cancer diagnosis (controls), using overall and stratified data by age, gender, race, and ADI. We used ADI as a socioeconomic marker (35), categorizing patients into low (bottom 20%) and high (top 20%) groups.

Finally, statistically significant associations with increased cancer risk were defined as RR >1 with 95% confidence intervals (CI) excluding 1 and a Benjamini–Hochberg false discovery rate (BH FDR)–adjusted *P* value < 0.05. Results were graphically presented using stacked and grouped bar plots, clustered heatmaps, and radial charts generated using Python libraries (RRID: SCR_008394) including Matplotlib (RRID: SCR_008624) and Seaborn (RRID: SCR_018132).

## Results

We aimed to identify which ICD-10 chapters and codes are associated with a subsequent cancer diagnosis using overall data and data stratified by age, gender, race, and ADI. In addition, we first performed a demographics analysis for the overall dataset (refer to Table 1) and the 20 most common types of cancer represented in our dataset (2, 36). The demographics of each group are listed in Tables 1 and 2.

**Table 1.** Demographics of individuals with cancer (%).

Variable	Overall	Patients with cancer	
		Yes	No
All patients	1,460,738	921,149 (63.06)	539,589 (36.94)
Age	47 ± 17.70	48.74 ± 17.80	43.95 ± 17.13
	​	t-value = 159.22; *P* value = 1e−09	
Age group	19–44	418,625 (57.92)	304,078 (42.08)
	45–64	297,096 (65.43)	156,984 (34.57)
	65–84	205,427 (72.35)	78,516 (27.65)
​	*χ* ^2^ value = 19,789.670; *P* value = 1e−09; df = 2		
Gender	​	​	​
Female	777,589 (53.22)	518,474 (66.68)	259,115 (33.32)
Male	682,795 (44.74)	402,614 (58.96)	280,181 (41.04)
Other	354 (2.04)	61 (17.23)	293 (82.77)
​	​	*χ* ^2^ value = 9,600; *P* value = 1e−09; df = 2	
Race	​	​	
White	925,705 (63.37)	588,610 (63.59)	337,095 (36.41)
Black or AA	420,282 (28.77)	283,441 (67.44)	136,841 (32.56)
Other	114,751 (7.86)	49,098 (42.78)	65,653 (57.22)
​	​	*χ* ^2^ value = 23,819.06; *P* value = 1e−09; df = 2	
Ethnicity/Hispanic	​	​	​
Yes	80,031 (5.48)	42,054 (52.55)	37,977 (47.45)
No	1,311,330 (89.77)	856,183 (65.29)	455,147 (34.71)
Other	69,377 (4.75)	22,912 (33.03)	46,465 (66.97)
​	​	*χ* ^2^ value = 33,466.19; *P* value = 1e−09; df = 2	
ADI	​	​	​
Low ADI	​	253,313 (66.45)	127,870 (33.55)
High ADI	​	56,533 (59.28)	38,829 (40.42)
​	​	*χ* ^2^ value = 1,724.74; *P* value = 1e−09; df = 1	

Abbreviations: AA, African American; df, degree of freedom.

**Table 2.** Characteristics and statistics for overall cancer and 20 cancer types.

Cancer subtype	Number of patients	Median age	Gender (%)		Race (%)		Hispanic (%)	
			Male	Female	White	Black/AA	No	Yes
Breast	3,642	66.71	0.40	99.60	69.21	28.63	96.24	2.90
Skin	3,364	69.93	58.72	41.28	97.53	01.71	98.17	1.71
Prostate	1,352	71.99	100	0	63.09	35.11	96.30	2.66
Lung	952	69.68	53.57	46.43	69.85	27,31	97.10	1.26
Colorectal	673	67.56	54.23	45,77	68.64	28.68	96.71	2.38
Liver	857	64.94	61.61	38.39	71.06	25.55	95.68	3.03
Bladder	410	71.79	70.98	20.02	79.02	17.80	96.09	1.71
Diffuse NHL	389	64.53	40.87	59.13	75.32	19.79	96.40	2.83
Leukemia	372	68.76	56.72	43.28	72.58	25.54	97.85	1.88
Kidney	323	67.05	63.78	36.22	72.58	25.38	95.67	3.10
Thyroid	292	57.86	21.92	78.76	78.77	17.12	93.84	4.11
Follicular NHL	291	62.79	35.74	64.26	74.91	20.27	96.56	3.09
Pancreatic	277	69.26	53.35	47.65	71.48	25.27	97.47	2.16
Endometrial	195	66.22	1.53	98.46	68.21	28.72	94.87	3.08
Ovarian	148	64.95	0	100	82.43	16.23	95.27	2.70
Stomach	127	69	59.84	40.16	59.06	40.16	94.48	4.72
Tongue	60	68.16	61.67	38.33	61.67	13.33	96.67	1.67
Tonsil	31	61.89	83.87	16.13	83.87	16.13	93.55	6.45
Anal	27	65.59	40.74	59.26	74.07	25.93	96.30	3.70
Oropharyngeal	8	61.36	62.5	37.5	87.5	12.5	100	0

### Overall data: Which ICD-10-CM chapters and codes are associated with a subsequent diagnosis of cancer or a cancer subtype?

For overall cancer, there was a significant association (RR >1, 95% CI excluding 1, and BH FDR–adjusted *P* < 0.05) with the following ICD-10 chapters: chapter 21 [health status factors; RR = 1.46 (1.45–1.47)], chapter 4 [metabolic; RR = 1.30 (1.26–1.32)], chapter 14 [genitourinary; RR = 1.30 (1.29–1.31)], chapter 3 [blood and immune disorder; RR = 1.19 (1.12–1.27)], chapter 18 [symptoms and signs; RR = 1.07 (1.06–1.08)], and chapter 13 [musculoskeletal; RR = 1.02 (1.01–1.03)]. We identified 221 ICD-10-CM codes significantly associated with increased cancer risk (RR >1, 95% CI excluding 1, and BH-FDR–adjusted *P* < 0.05). The top five were systemic sclerosis [M34; RR = 5.27 (4.86–5.68)], blood type [Z67; RR = 5.16 (4.81–5.51)], benign mammary dysplasia [N60; RR = 437 (3.92–4.83)], immune mechanism [D89; RR = 4.33 (3.86–4.80)], and disturbances of smell/taste [R43; RR = 4.31 (4.06–4.57)]. The RR of other notable PHCs were lipoprotein metabolism disorders [E78; RR = 2.76 (2.65–2.87)], diabetes [E10 (type 1); RR = 2.94 (2.79–3.09) and E11 (type 2); RR = 1.79 (1.74–1.84)]. Human immunodeficiency virus [HIV; B20; RR = 1.81 (1.64–1.99)], vitamin D deficiency [E55; RR = 1.65 (1.57–1.73)], abnormal tumor markers [R97; RR = 2.10 (2–2.20)], heart failure [I50; RR = 1.36 (1.26–1.46)], and cicatricial alopecia [L66; RR = 1.30 (1.14–1.46)] increase overall/subtype cancer risk within 5 years.

ICD-10 chapters linked to specific cancers were identified across 20 cancer types. Supplementary Figure S2 illustrates a stacked bar diagram showing the overall RR and RR for 20 cancer subtypes. Across more than 20 cancer subtypes, chapters with a median RR (MRR) greater than 1 included chapters 12 (skin diseases), 9 (circulatory diseases), 14, 11, 4, and 13, with MRRs of 2 to 58, 2 to 39, 1 to 79, 1 to 43, 1 to 42, and 1 to 14, respectively. Chapters 9 and 17, though not overall risk factors, were significantly associated with 13 and 1 specific cancer types, respectively. Chapters 1, 4, 9, 14, 12, and 17 were associated with elevated cancer risks, each linked to 5, 13, 14, 12, 10, and 1 specific types, respectively.

**Figure 2.**
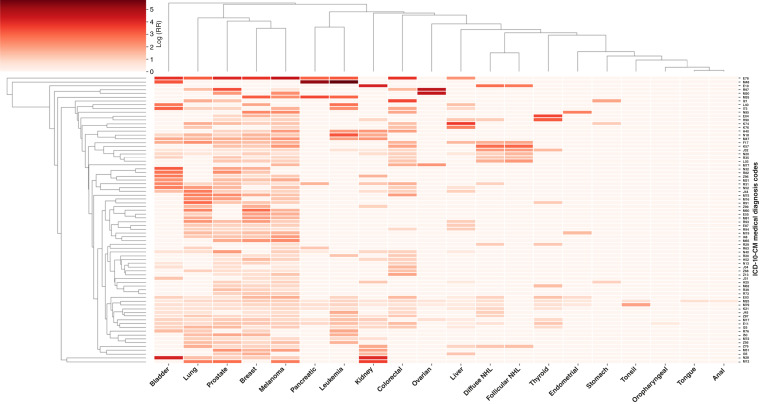
A hierarchical clustering heatmap of the top 76 ICD-10-CM codes (RR >1 in >3 subtype cancers) grouped by 20 cancer types. Darker red indicates higher risk. Lung, prostate, breast, and skin cancers emerge as key early cancer indicators, strongly associated with multiple conditions.

As illustrated in Fig. 2, clustering of 76 high-risk codes (RR >1 in >3 subtype cancers) revealed shared patterns, with lung, prostate, breast, and skin cancers strongly associated with type 2 diabetes (E11), disorders of lipoprotein metabolism (E78), nicotine dependence (F17), blood in urine (hematuria, R31), and lifestyle/preventive care (Z76). Type 2 diabetes is associated with 11 cancer subtypes, including colorectal, bladder, skin, pancreatic, prostate, lung, and breast cancers and leukemia. Disorders of lipoprotein metabolism are associated with 10 cancer subtypes, including skin, breast, lung, bladder, and prostate cancers. Nicotine dependence is associated with 10 cancer subtypes, including lung cancer, skin cancer, follicular non–Hodgkin lymphoma (NHL), and diffuse NHL. Hematuria is associated with 10 cancer subtypes, including colorectal, bladder, pancreatic, and prostate cancers. Individuals encountering health services are associated with 9 cancer subtypes, including kidney cancer, lung cancer, diffuse NHL, follicular NHL, and breast cancer. Additional signals included vascular occlusions, angina, diverticular disease, spondylopathies, benign prostatic hyperplasia, systemic sclerosis, and abnormal tumor markers, highlighting key early indicators across.

### Stratified data: What factors influence which ICD chapters and codes associate with a subsequent diagnosis of cancer?

A similar analysis using truncated ICD-10-CM codes was conducted. This generated a clustered heatmap of 55 codes, each showing at least 1 RR greater than 2 with a statistically significant CI across 3 age groups, as shown in Supplementary Fig. S3. This figure highlights the top cancer risk factors by age group. For ages 19 to 44, major risk factors included disturbances of smell and taste (R43; RR = 9.64), type 1 diabetes (E10; RR = 3.59), abnormal menstrual cycles (N91; RR = 2.86), sickle cell disorders (D57; RR = 2.86), and benign mammary dysplasia (N60; RR = 2.49). For ages 45 to 64, major risk factors included HIV disease (B20; RR = 5.22), vaginal disorders (N89; RR = 3.86), unspecified lump in the breast (N63; RR = 3.62), and other breast disorders (N64, RR = 3.39). Among individuals 65 to 84 year of age, multiple sclerosis (G35; RR = 7.07), testicular failure (E29; RR = 5.40), and lipoprotein metabolism disorders (E78; RR = 4.63) were identified as major risk factors.

**Figure 3.**
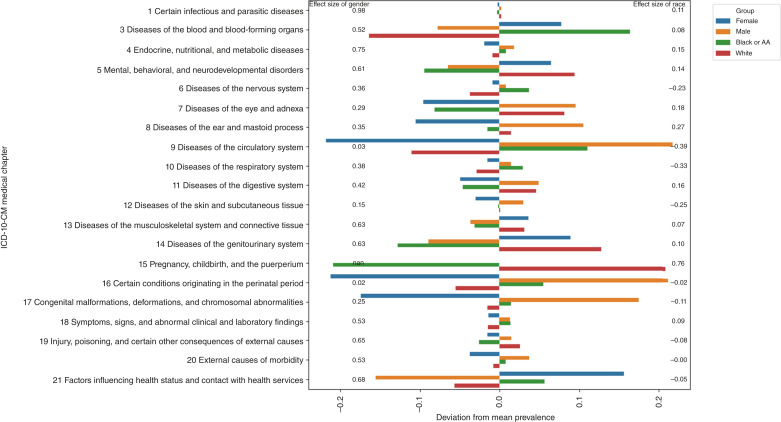
Group barplots depicting the deviation from mean RR by gender and race with annotated effect sizes for 20 ICD-10-CM chapters across all patients. Overall, these effect sizes indicate that cancer affects women more than men and that cancer experiences differ between Black individuals and White individuals. These insights are crucial for tailoring cancer research to meet the specific needs of various groups. AA, African Americans.

Next, we analyzed prevalence, calculated Cohen d effect sizes, and RR across all chapters, comparing females with males and White with Black individuals and in four combinations [Black male (BM), White male (WM), Black female (BF), and White female (WF)]. Gender and racial effect size differences are illustrated in Fig. 3. Male showed strong to medium effect sizes in chapters 1, 3, 4, 5, 13, 14, and 21, whereas female exhibited strong to medium effect sizes in chapters 3, 5, 13, 14, and 21. A medium effect size was observed for Black compared with White individuals in chapter 9. In RR analysis, WF individuals have higher RRs than BF individuals across most ICD-10 chapters, except for chapter 3, in which BF individuals show a significantly higher risk (RR = 1.01 vs. 1.86). BM individuals tend to have the highest risks overall, particularly in chapters 4 (RR = 1.79) and 12 (RR = 1.21), whereas WM individuals have the highest risks in chapters 1 (RR = 1.05) and 14 (RR = 1.57).

For gender stratification, 348 PHCs (males) and 310 health conditions (HC; females) with RR >1 were identified. The top conditions for males were disturbances of smell/taste (R43; RR = 8.81), skin changes (L57; RR = 7.81), hypertensive chronic kidney disease (I12; RR = 7.08), rheumatoid arthritis (M05; RR = 5.13), seborrheic keratosis (L82; RR = 4.87), lipoprotein metabolism disorders (E78; RR = 5.93), and type 1 diabetes (E10; RR = 4.86). For females, the top conditions were disturbances of smell/taste (R43; RR = 5.76), systemic sclerosis (M34; RR = 5.71), blood type (Z67; RR = 5.69), HIV disease (B20; RR = 4.06), immune mechanism (D89; RR = 4.52), benign mammary dysplasia (N60; RR = 4.26), osteoarthritis (M18; RR = 3.85), and lipoprotein metabolism disorders (E78; RR = 3.57). For race stratification, we identified 357 PHCs (Black) and 325 HCs (White) with RR >1. The top conditions for Black people were disturbances of smell/taste (R43; RR = 7.98), blood type (Z67; RR = 6.74), benign mammary dysplasia (N60; RR = 5.84), type 1 diabetes (E10; RR = 4.11), lipoprotein metabolism disorders (E78; RR = 3.98), immunodeficiencies (D84; RR = 3.59), hypertensive heart disease (I11; RR = 3.43), and ankylosing spondylitis (M45; RR = 3.39). For White people, the top conditions were lipoprotein metabolism disorders (E78; RR = 5.90), disturbances of smell/taste (R43; RR = 5.52), abnormal tumor markers (R97; RR = 5.49), organ or tissue transplant (Z94; RR = 4.80), blood type (Z67; RR = 4.77), and endometriosis (N80; RR = 4.64).

A hierarchical clustering heatmap of 98 PHCs (ICD-10-CM codes, RR >2) across four demographic groups is presented in Supplementary Fig. S4. Type 1 diabetes (E10; RRs for BM = 4.68; BF = 3.54; WM = 4.56; WF = 2.58) is the only PHC strongly linked across all groups, whereas lipoprotein metabolism disorders (E78; RRs for BM = 5.22; WM = 4.04; BF = 3.30; WF = 0) are common in 3 groups. Males in both racial groups show strong associations with 10 PHCs, including abnormal tumor markers (R97; RRs for Black = 3.19; White = 10.18), viral hepatitis (B19; RRs for Black = 2.89; White = 5.42), benign prostatic hyperplasia (N40; RRs for Black = 3.40; White = 10.48), type 1 diabetes (E10; RRs for Black = 4.67; White = 4.56), type 2 diabetes (E11; RRs for Black = 3.40; White = 3.12), and Crohn disease (K50; RRs for Black = 2.69; White = 3.16). Females show 5 distinct PHCs, including chlamydial infections (A74; RRs for Black = 3.5; White = 3.77), bacterial infections (B96; RRs for Black = 2.80; White = 2.67), rare menstruation (N91; RRs for Black = 2.78; White = 2.89), and communicable diseases (Z20; RRs for Black = 2.81; White = 2.93). Race-specific PHCs for Black individuals included disturbances of smell and taste (R43; RRs for male = 14.06; female = 5.70), chronic inflammatory disease (M45; RRs for Black = 3.53; White = 3.42), and health services (Z76; RRs for male = 4.26; female = 2.87). For White people, race-specific PHCs included nicotine dependence (F17; RRs for male = 2.71; female = 2.92), HIV disease (B20; RRs for male = 2.53; female = 3.17), and multiple sclerosis (G35; RRs for male = 4.74; female = 2.64). For each group, we identified cancer risk factors: 37 for BMs [e.g., disturbances of smell and taste (R43; RR for male = 14.06; female = 5.70), chronic radiation skin changes (L57; RR = 7.51), kidney disease (I12; RR = 6.01)]; 38 for WMs [e.g., abnormal tumor markers (R97; RR = 10.18), viral hepatitis (B19; RRs for Black = 2.89; White = 5.42), and heart failure (I50; RR = 4.77)]; 27 for BFs [e.g., blood type (Z67; RR = 6.21) and benign mammary dysplasia (N60; RR = 4.95)]; and 26 for WFs [e.g., symptoms and signs (R39; RR = 4.35) and cicatricial alopecia (L66; RR = 4.41)].

Finally, we analyzed the impact of socioeconomic factors on cancer diagnoses (Supplementary Figs. S5 and S6). The hierarchical clustering heatmap (RR >2) groups 62 ICD-10-CM codes by low and high ADI. Nine conditions, including type 1 diabetes (E10; low RR = 4.37; high RR = 5.49) and various urinary issues (R39; low RR = 2.74; high RR = 5.70), show high cancer risk in both groups. Thirteen conditions, including disturbances of smell/taste (R43; low RR = 6.5; high RR = 0), inflammatory liver diseases (K75; low RR = 5.30; high RR = 0), lipoprotein metabolism disorders (E78; low RR = 5.33; high RR = 0), and breast conditions (N64; low RR = 4.40; high RR = 0), are linked to low ADI, whereas 47 conditions, including testicular dysfunction (E29; low RR = 1.39; high RR = 5.60), HIV disease (B20; low RR = 1.19; high RR = 6.77), opioid use (F11; low RR = 0.64; high RR = 3.42), are linked to high ADI.

**Figure 4.**
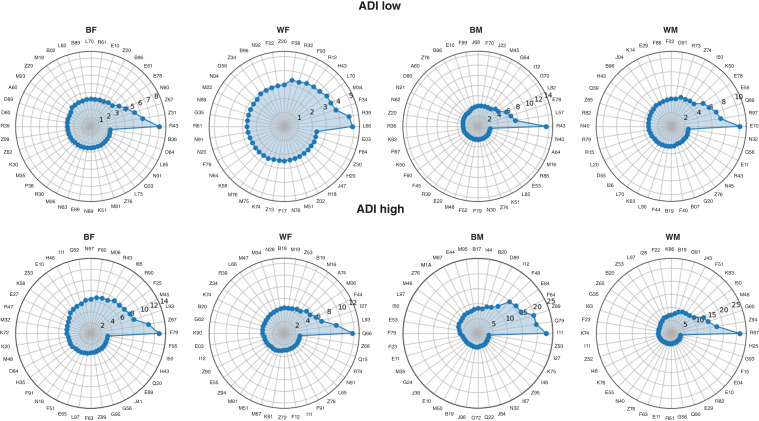
Radial plots of ADI-, gender-, and race-stratified analyses clearly show that HCs associated with cancer risk vary across ADI levels, gender, and race. Among BF patients, disturbances of smell and taste (R43) was the top predictor at low ADI, whereas unspecified intellectual disability dominated (F79) at high ADI. In WF patients, cicatricial alopecia (L66) was most predictive at low ADI, whereas congenital deformities of feet (Q66) was most predictive at high ADI. For BM patients, R43 ranked highest at low ADI, but hypertensive heart disease (I11) was most predictive at high ADI. In WM patients, type 1 diabetes (E10) was the leading predictor at low ADI, whereas abnormal tumor markers (R97) emerged as the strongest predictor at high ADI for developing overall cancer within 5 years.

In addition, our ADI-, gender-, and race-stratified analyses clearly show that HCs associated with cancer risk vary across ADI levels, gender, and race (Fig. 4). Among BF patients, disturbances of smell and taste (R43) was the top predictor at low ADI, whereas unspecified intellectual disability dominated (F79) at high ADI. In WF patients, cicatricial alopecia (L66) was most predictive at low ADI, whereas congenital deformities of feet (Q66) was most predictive at high ADI. For BM patients, R43 ranked highest at low ADI, but hypertensive heart disease (I11) was most predictive at high ADI. In WM patients, type 1 diabetes (E10) was the leading predictor at low ADI, whereas abnormal tumor markers (R97) emerged as the strongest predictor at high ADI for developing overall cancer within 5 years. Results for all 20 cancer types, including the full dataset and subgroup analyses by gender, race, and ADI, are shown in the Supplementary Cancer-Specific Results and Supplementary Table S2.

Based on all these findings, we developed a rule-based deterministic framework to predict cancer susceptibility by identifying pre-HCs associated with subsequent cancer within 5 years (see Supplementary Fig. S7).

## Discussion

Our study provides a comprehensive analysis of PHCs associated with cancer risk within the next 5 years using large-scale EHR data, stratified by age, gender, race, and socioeconomic factors. The statistical tests allowed us to identify significant differences in these variables between those who developed cancer and those who did not, with a *P* value < 1e−10. At least one diagnosis within a particular ICD chapter (e.g., health status factors, metabolic, genitourinary, and blood and immune disorder) carries an increased risk of subsequently being diagnosed with one or more cancer types within 5 years. In addition, 221 ICD-10-CM medical diagnosis codes were found to be significantly associated with an increased risk of at least 1 of 20 common cancer types. Although chapter 9 was not associated with an increased overall cancer risk, it was significantly linked to a higher risk of 13 specific cancer types. This suggests that increases observed in certain chapters may have been offset by decreases in others when considering overall cancer incidence.

Across the 3 age groups, abnormal menstrual cycles (N91) are linked to a higher cancer risk. However, type 1 diabetes (E10) and disturbances of smell and taste (R43) are high-risk factors primarily among individuals 19 to 44 years of age. For those 45 to 64 years of age, conditions such as HIV disease (B20), breast disorders (N64), lipoprotein metabolism disorders (E78), and unspecified breast lump (N63) are associated with an increased cancer risk. Among individuals 65 to 84 years of age, the highest risk is observed for multiple sclerosis (G35) and testicular failure (E29).

There were marked gender-related, race-related, and socioeconomic factor–related differences in RRs associated with truncated ICD codes. Differences in cancer risk related to an ICD chapter were more pronounced for gender than for race. For example, female subgroups exhibited higher deviations in chapters 3, 5, 13, and 14, whereas male subgroups showed notable deviations in chapters 1, 4, 10, 11, 18, and 21. This finding suggests that factors deriving from sex may be associated with differing susceptibility to certain conditions and diseases. Black individuals displayed pronounced deviations in chapters 4, 5, 9 and 21, suggesting that race, in turn, is associated with different susceptibilities compared with gender. It is important to note that these susceptibilities may arise from cultural and behavioral associations rather than genetic factors. Similarly, chapters 3 and 14 show a predominance among White individuals, suggesting occupational hazards or lifestyle factors that may be more common in White populations. Cohen d effect size analysis highlighted substantial gender disparities in chapters 1 (d = 1.02), 3 (d = 0.54), 4 (d = 0.48), and 14 (d = 0.63), whereas smaller but meaningful race disparities were observed in chapter 9 (d = −0.04). In PHCs, high-risk conditions such as disturbances of smell and taste (R43), disorders of lipoprotein metabolism (E78), blood type (Z67), osteoarthritis (M18), benign mammary dysplasia (N60), type 1 diabetes (E10), and abnormal tumor markers (R97) emerged as top cancer risk factors across multiple subgroups. These findings underscore the importance of targeted screening and prevention strategies tailored to address demographic-specific cancer risks.

Our ADI results indicate similar associations with socioeconomic factors. In individuals with a low ADI, PHCs like liver diseases (K75), lipoprotein metabolism disorders (E78), and breast conditions (N64) were prevalent, whereas in people with a high ADI, testicular dysfunction (E29), HIV disease (B20), and opioid use (F11) posed significant risks. These findings emphasize the impact of socioeconomic factors on cancer risk, with distinct conditions contributing to disparities in cancer prevalence between individuals in affluent and deprived groups. An alternate explanation is that individuals with low ADI, those with the least deprivation and greater access to health insurance, interact with the healthcare system with greater frequency, allowing earlier, less acute presentation of indicators of later cancer. Regardless, ADI emerged as a statistically significant mediating variable in our analysis.

Several identified associations warrant cautious interpretation. Certain ICD groupings may reflect shared etiologic exposures (e.g., tobacco use underlying both chronic obstructive pulmonary disease and lung cancer) or increased diagnostic intensity due to imaging-triggering conditions, rather than direct causal pathways. These findings should therefore be interpreted as associative patterns within real-world healthcare data rather than mechanistic risk factors.

Many findings align with prior research, with benign mammary dysplasia and abnormal breast imaging supporting early cancer diagnosis, thereby validating our methodology (36). Systemic sclerosis and smell/taste disturbances were unexpected but not novel. Weeding and colleagues (37) reported that patients with scleroderma have a 1.5–4× higher cancer risk, with cancer occurring concurrently or years later, suggesting shared etiology or treatment effects. Similarly, Mascaritoli and colleagues (2017) found that malnutrition, anorexia, and weight loss are common among patients with cancer before oncology visits, implying that some cancers may impair smell and taste and contributing to malnutrition. Our findings confirm the link between systemic sclerosis and cancer, particularly breast, lung, and skin cancers, aligning with prior research (38). Lipoprotein metabolism disorders significantly correlate with multiple cancers, including breast, skin, prostate, colorectal, bladder, pancreatic, endometrial, and tongue cancers and NHL. As noted, these disorders are linked to hyperlipidemia, lipid storage disease, and obesity, warranting further study on their molecular role in cancer (39). Kidney disease, the second leading cause of death in patients with cancer, is associated with bladder, kidney, anal, and oropharyngeal cancers in this study (40).

These findings support the development of a rule-based artificial intelligence (AI) tool that predicts cancer susceptibility and could ultimately enable personalized risk models capable of flagging high-risk patients and informing targeted screening recommendations (Supplementary Fig. S7). This graphical user interface of the rule-based AI system developed for 5-year cancer susceptibility estimation. The interface allows users to input demographic characteristics (age group, gender, and race), socioeconomic context (ADI), and PHCs (ICD codes). Upon submission, the rule engine evaluates predefined if–then logic rules derived from epidemiologic associations and computes RR contributions for each triggered condition. The final cancer susceptibility score is generated through deterministic rule aggregation without any machine learning training procedure. The system provides full transparency by displaying individual ICD-specific risk contributions and the overall 5-year susceptibility estimate. In future work, we will enhance this AI tool using multidisease pathway analysis, 8 interpretable machine learning models, and 4 large language models to extract temporal and contextual cancer-risk insights from EHR data based on 1,229 binary ICD-10-CM features and sequential patient histories. The RR-based findings are intended to identify high-risk groups, inform hypothesis generation, and support future predictive modeling frameworks.

### Limitations

Data were collected from multiple hospitals within a state-wide hospital system and may not be representative of patients at other locations. Unsupervised analysis prevents causal conclusions. Although detailed analysis provides insights into early detection and understanding of cancer progression, a longitudinal study is needed to map precancerous pathways across cancer types, regions, and demographics. In addition, ADI may not fully reflect individual-level socioeconomic or environmental exposures, and the lack of tobacco data may introduce residual confounding. Future work should incorporate additional environmental and behavioral factors (e.g., tobacco use) and external validation across health systems to enhance generalizability and enable translation to patient-level predictive models.

## Supplementary Material

Appendix Supplementary Appendix: Cancer-Specific Results

Supplementary Materials Supplementary Appendix: Materials and Methods

Supplementary Table S1 Table S1. Standard and binomial relative risks of ICD-10-CM medical diagnosis chapters across all patients.

Supplementary Table S2 Table S2. Top selected ICD-10-CM medical diagnoses across overall, gender, race, gender-race, and ADI patient groups.

Supplementary Figure S1 Figure S1. CONSORT diagram summarizing the data inclusion and exclusion process.

Supplementary Figure S2 Figure S2. Stacked barplots depicting the relative risk of overall cancer and 20 specific cancer types, categorized by 20 ICD-10-CM Chapters.

Supplementary Figure S3 Figure S3. A clustered heatmap of 55 codes, each with at least one RR greater than 2.0 (with a significant confidence interval) across three age groups.

Supplementary Figure S4 Figure S4. A hierarchical clustering heatmap of 98 ICD-10-CM codes (RR >2.0) across four demographic groups.

Supplementary Figure S5 Figure S5. Radial plot comparing relative risks (RR) for 20 cancer types between low-ADI and high-ADI populations.

Supplementary Figure S6 Figure S6. A clustered heatmap of socioeconomic factors' impact on cancer diagnoses in low and high ADI.

Supplementary Figure S7 Figure S7. Graphical user interface (GUI) of the rule-based artificial intelligence system developed for 5-year cancer susceptibility estimation.

## Data Availability

All analytic results, derived data files, and code are available at https://github.com/MAAlam79/AI-Cancer-PHC-Risk. Raw EHR data cannot be shared publicly because of patient privacy and institutional restrictions but may be accessed upon reasonable request and appropriate approvals.
