# Supplementary material for: Association between Diagnostic History and Cancer Incidence within 5 Years: A Real-world Observational Analysis
Source: Cancer Res Commun. 2026 May 11;6(5):1083–91. doi: 10.1158/2767-9764.CRC-26-0163 (PMC13158651; doi:10.1158/2767-9764.CRC-26-0163)
Supplement: Appendix — Supplementary Appendix: Cancer-Specific Results [file crc-26-0163_appendix_suppsm.docx]

Supplementary Appendix: Cancer-Specific Results

Association between diagnostic history and cancer incidence within 5 years: a real-world observational analysis

Md Ashad Alam^1,2^, Grace Williams^1^, Muhammad G. Kibriya^3^, Marc Matrana^2,4^, Nick Duesbery^1,2^, Edward Trapido^2,5^, Daniel Fort^1^

^1^Ochsner Center for Outcomes Research, Ochsner Research, Ochsner Clinic Foundation, New Orleans, LA 70121, USA

^2^Louisiana Cancer Research Center, New Orleans, LA 70112, USA

^3^Public Health Sciences, Biological Sciences Division, University of Chicago, Chicago IL 60637, USA

^4^Ochsner MD Adnerson Cancer Center, Ochsner Health, New Orleans, LA 70112, USA

^5^Louisiana State University School of Public Health, New Orleans, LA 70112, USA

**Supplementary Appendix: Cancer Specific Results**

**Cancer Specific Results**

In addition to presenting the complete results in the supplementary files, we have summarized key findings in Table S2. This table highlights the top ICD-10-CM medical diagnosis codes for 20 different types of cancer across various demographics, including overall data, gender, race, gender-race combinations, and ADI-specific data. Notable results are highlighted for each type of cancer below.

For **breast cancer,** Table S2 reveals that the code [other disorders of breast](https://icd.codes/icd10cm/N64) (N64), appears four times, and R92 appears three times out of 11 instances associated with an increased risk of breast cancer in the overall data. For white individuals, HCs unspecified lump in the breast (N63), [other disorders of breast](https://icd.codes/icd10cm/N64) (N64), and other nontoxic goiter (E04) suggest a higher risk of breast cancer. Additionally, N60 and [acute pancreatitis](https://icd.codes/icd10cm/K85) (K85) are identified as risk factors for the development of breast cancer in prosperous conditions, while R67 and other hypothyroidism (E03) are risk factors in poverty.

**For skin cancer,** L57 and fracture of shoulder and upper arm (S42) appear in 3 out of 11 situations, indicating an increased risk of skin cancer in the overall data. Specifically, L57 is linked to a higher risk in male individuals, while R92 and S42 are associated with a higher risk in females. For black individuals, M34, E87, and M48 are prominent risk factors, while for white individuals, O02, H40, and O20 are significant. Notably, L57 is associated with a higher risk in male black individuals, while D62 is linked to female black individuals. Additionally, L57 is a risk factor for male white individuals, while R47 is for female white individuals. Moreover, R97 and R47 are identified as risk factors for skin cancer in prosperous conditions, while N50 and N40 are risk factors in poverty.

For **prostate cancer**, R97, L28, and E78 appear in 6, 3, and 3 out of 11 situations, respectively, indicating an increased risk of prostate cancer in the overall data. For Male-Black individuals, R97 and L28 are prominent risk factors, while for Male-White individuals, R97 and E78 are significant. Additionally, R97 and H16 are identified as risk factors for prostate cancer in prosperous conditions, while R93 and N40 are risk factors in poverty.

For **lung cancer**, H02 and K74 appear in 6 and 3 out of 11 situations, respectively, along with M37 and L97, indicating an increased risk of developing lung cancer in the overall data. Specifically, M21, M75, and R97 are linked to a higher risk in male individuals, while H02, I21, and N18 are associated with a higher risk in females. For Black individuals, H02, I21, and R97 are prominent risk factors, while for white individuals, K74, I71, and R97 are significant. Notably, M94, M12, and H26 are associated with a higher risk in male black individuals, while H02, H80, and L90 are linked to female Black individuals. Additionally, I71, M75, and K74 are risk factors for male white individuals, while N18, J44, and F17 are for female white individuals. Moreover, Z76 and H02 are identified as risk factors for developing lung cancer in prosperous conditions, while H00 and M25 are risk factors in poverty.

For **colorectal** cancer, L97 and F03 appear in 4 and 3 out of 11 situations, respectively, along with I21 and E78, indicating an increased risk of developing colorectal cancer in the overall data. Specifically, F03, J45, and E03 are linked to a higher risk in male individuals, while R32, N18, and L97 are associated with a higher risk in females. For black individuals, L97, K46, and N18 are prominent risk factors, while for white individuals, F03, G47, and R50 are significant. Notably, J45, E03, and J31 are associated with a higher risk in male Black individuals, while R50 and G47 are linked to female black individuals. Additionally, N18, R32, and L97 are risk factors for male White individuals, while H04 and M79 are for female White individuals. Moreover, N13 and F03 are identified as risk factors for developing colorectal cancer in prosperous conditions, while I73 is a risk factor in poverty.

For **liver** cancer, K74 and K76 appear in 10 and 3 out of 11 situations, respectively, along with I85 and K70, indicating an increased risk of developing liver cancer in the overall data. Specifically, I37, K74, and K76 are linked to a higher risk in male individuals, while I85, K74, and K76 are associated with a higher risk in females. For black individuals, I37, K74, and N41 are prominent risk factors, while for white individuals, K74 and K76 are significant. Notably, N32, J47, and K74 are associated with a higher risk in male black individuals, while K74 and H04 are linked to female black individuals. Additionally, I84, K74, and K72 are risk factors for male white individuals, while K74, K76, and D86 are for female white individuals. Moreover, K74 and K70 are identified as risk factors for developing liver cancer in prosperous conditions, while K74 and M25 are risk factors in poverty.

For **bladder cancer**, N28 appears in 5 out of 11 situations, along with M48 and E78, indicating an increased risk of developing bladder cancer in the overall data. Specifically, T84, N28, and J33 are linked to a higher risk in male individuals, while M48, L40, and I73 are associated with a higher risk in females. For black individuals, N28, R84, and L40 are prominent risk factors, while for white individuals, M21 and B35 are significant. Notably, N28, T84, and J33 are associated with a higher risk in male black individuals, while M12, B35, and E29 are linked to female black individuals. Additionally, K80, I73, and R76 are risk factors for male white individuals, while R31, M76, and L84 are for female white individuals. Moreover, K12 and N28 are identified as risk factors for developing bladder cancer in prosperous conditions, while J40 and I70 are risk factors in poverty.

For **diffuse NHL** cancer, B02 and E10 appear in 3 out of 11 situations, along with H34 and K58, indicating an increased risk of developing diffuse NHL cancer in the overall data. Specifically, E10, M35, and K22 are linked to a higher risk in male individuals, while I20, K57, and B02 are associated with a higher risk in females. For Black individuals, E10, B02, and I20 are prominent risk factors. Notably, J32, R10, and E78 are associated with a higher risk in male black individuals. Additionally, I20, B02, and K57 are risk factors for male white individuals, while O26, N89, and J32 are for female white individuals. Moreover, N89 and I70 are identified as risk factors for developing diffuse NHL cancer in prosperous conditions, while K44 is a risk factor in poverty.

For **leukemia cancer**, L93, M48, and E70 indicate an increased risk of developing the leukemia cancer in the overall data. Specifically, M71, I20, and H40 are linked to a higher risk in males, while L40, M38, and I73 are associated with a higher risk in females. Among black individuals, L40, M35, and I73 are prominent risk factors. Notably, M17, H40, and L20 are linked to a higher risk in black males. Additionally, L40, E70, and M67 are risk factors for white males, while J45, H40, and N18 are significant for white females. Moreover, J01 and T50 are identified as risk factors for developing bladder cancer in prosperous conditions, whereas H40 is a risk factor in poverty.

For **kidney cancer**, N28 and H17 appear in 7 and 4 out of 11 situations, respectively, along with M12, indicating an increased risk of developing kidney cancer in the overall data. Specifically, H17, N28, and H35 are linked to a higher risk in males, while N28, R53, and H90 are associated with a higher risk in females. Among black individuals, H17, N28, and H40 are prominent risk factors. Notably, H17, N28, and M50 are associated with a higher risk in Black males. Moreover, N28 and M12 are identified as risk factors for developing bladder cancer in prosperous conditions, while H40 and J70 are risk factors in poverty.

For **thyroid cancer**, E04 appears in 5 out of 11 situations, along with A31 and E21, indicating an increased risk of developing the disease in the overall data. Specifically, E04, F24, and E11 are linked to a higher risk in male individuals, while E05, G20, and R91 are associated with a higher risk in females. For black individuals, A31, E05, and E04 are prominent risk factors, while G20 and F25 are significant for white individuals. Notably, E04, E11, and R35 are associated with a higher risk in male Black individuals. Additionally, M47, F07, and J41 are risk factors for male White individuals, while N83, R13, and R49 are for female white individuals. Moreover, H35 and E04 are identified as risk factors for developing thyroid cancer in prosperous conditions, while F33 is a risk factor in poverty.

For **Follicular NHL** cancer, indicating an increased risk of developing Follicular NHL cancer in the overall data. Specifically, E10, M25, and R10 are linked to a higher risk in male individuals, while I20, J01 and K57 are associated with a higher risk in females. For black individuals, E10, I20 and K57 are prominent risk factors, while for white individuals, N20, O26 and N89 are significant. Notably, R10, E78, and H52 are associated with a higher risk in male black individuals. Additionally, I10, K57, and R35 are risk factors for male white individuals, while O26 and N89 are for female white individuals. Moreover, N87 and I70 are identified as risk factors for developing bladder cancer in prosperous conditions, while K44 are risk factors in poverty.

For **pancreatic cancer**, K86 appears in 3 out of 11 situations, along with M48 and G35, indicating an elevated risk of developing the cancer in the overall data. Specifically, K86, K64, and M77 are linked to a higher risk in male individuals, while E78, F41, and F43 are associated with a higher risk in females. Among black individuals, K86, R31, and K21 are prominent risk factors, whereas M48, G35, and F41 are significant for white individuals. Notably, K85, K64, and K21 are associated with a higher risk in male black individuals, while N51 and M47 are linked to female black individuals. Furthermore, M77, R31, and E78 are risk factors for male white individuals, while M25, M79, and M17 are for female white individuals. Additionally, K82 and G24 are identified as risk factors for developing pancreatic cancer in prosperous conditions, while G60 and M17 are risk factors in poverty.

For **endometrial cancer**, N95 and F22 appear in 4 and 3 out of 11 situations, respectively, along with E78, indicating an increased risk of developing this type of cancer in the overall data. Specifically, L97 is linked to a higher risk in male individuals, while F22, G47, and N65 are associated with a higher risk in females. Among black individuals, K34, N95, and G47 are prominent risk factors. Additionally, G74 and B30 are identified as risk factors for developing endometrial cancer in prosperous conditions.

For **ovarian cancer**, M77 appears in three out of 11 instances, along with R97, M50, and N83, indicating an increased risk of developing this cancer in the overall data. Specifically, M78, M77, and F25 are linked to a higher risk in female individuals. Among black individuals, M76, M77, and H52 are prominent risk factors, while F35 is significant for white individuals. Furthermore, M76, M77, and E04 are risk factors for female black individuals, while F43, F41, and N83 are for female white individuals. Additionally, F32, F41, and N83 are identified as risk factors for developing ovarian cancer in prosperous conditions, while G60 and M17 are risk factors in poverty.

For **stomach cancer**, F44 and J38 appear in 4 and 3 out of 11 instances, respectively, along with R49 and I21, indicating an increased risk of developing this type of cancer in the overall data. Specifically, F46, M95, and M51 are linked to a higher risk in male individuals, while F44, R49, and J38 are associated with a higher risk in females. Among black individuals, F44, J38, and R49 are prominent risk factors, whereas J33 and K92 are significant for white individuals. Notably, F44, J38, and R49 are associated with a higher risk in female black individuals. Additionally, J33 and A09 are identified as risk factors for developing bladder cancer in prosperous conditions, while M51 is a risk factor in poverty.

For **tongue cancer**, M10 appears in 4 out of 11 instances, along with E78, indicating an increased risk of developing this type of cancer in the overall data. Specifically, M10 is linked to a higher risk in male individuals, while F46, N94, and H92 are associated with a higher risk in females. Among black individuals, M25 is a prominent risk factor, whereas M65, M10, and M19 are significant for white individuals. Notably, E78 and L03 are associated with a higher risk in male black individuals, while M10 is linked to female black individuals. Additionally, H59 and H91 are identified as risk factors for male white individuals, while J44 is significant for female white individuals. Moreover, M25 and M79 are identified as risk factors for developing bladder cancer in prosperous conditions.

For **tonsil cancer**, M79 appears in 4 out of 11 situations, indicating an increased risk of developing this type of cancer in the overall data. Specifically, M79 is linked to a higher risk in male individuals, while H59 and H91 are associated with a higher risk in females. Among black individuals, M75 and M25 are prominent risk factors, with M79 being particularly significant for male black individuals. Additionally, H59 and H91 are identified as risk factors for male white individuals. Moreover, M25 and M79 are recognized as risk factors for developing bladder cancer in prosperous conditions.

For **anal cancer**, M25 appears in 3 out of 11 situations, along with K50, K62, and M72, indicating an increased risk of developing anal cancer in the overall data. Specifically, M12, Q66, and M25 are linked to a higher risk in male individuals, while N28, F33, and R03 are associated with a higher risk in females. Among black individuals, M25 and N28 are prominent risk factors, while Q66, G57, and M19 are significant for white individuals. Additionally, M25 and M77 are identified as risk factors for developing anal cancer in prosperous conditions.

For **oropharyngeal cancer**, E01 is identified as indicating an increased risk of developing this type of cancer in the overall data. Specifically, F11 is linked to a higher risk in male individuals. Among black individuals, K43 is a prominent risk factor, while E01 is significant for white individuals. Additionally, N28 is recognized as a risk factor for developing oropharyngeal cancer in prosperous conditions.
