# Supplementary material for: Association between Diagnostic History and Cancer Incidence within 5 Years: A Real-world Observational Analysis
Source: Cancer Res Commun. 2026 May 11;6(5):1083–91. doi: 10.1158/2767-9764.CRC-26-0163 (PMC13158651; doi:10.1158/2767-9764.CRC-26-0163)
Supplement: Supplementary Materials — Supplementary Appendix: Materials and Methods [file crc-26-0163_supplementary_materials_suppsm.docx]

Supplementary Appendix: Materials and Methods

Association between diagnostic history and cancer incidence within 5 years: a real-world observational analysis

Md Ashad Alam^1,2^, Grace Williams^1^, Muhammad G. Kibriya^3^, Marc Matrana^2,4^, Nick Duesbery^1,2^, Edward Trapido^2,5^, Daniel Fort^1^

^1^Ochsner Center for Outcomes Research, Ochsner Research, Ochsner Clinic Foundation, New Orleans, LA 70121, USA

^2^Louisiana Cancer Research Center, New Orleans, LA 70112, USA

^3^Public Health Sciences, Biological Sciences Division, University of Chicago, Chicago IL 60637, USA

^4^Ochsner MD Adnerson Cancer Center, Ochsner Health, New Orleans, LA 70112, USA

^5^Louisiana State University School of Public Health, New Orleans, LA 70112, USA

**Relative Risk**

RR is a measure used to compare the risk of a certain event (such as developing cancer) occurring in case group to the risk of the same event occurring in a control group. It is calculated as the ratio of the probability of the event occurring in the case group to the probability of the event occurring in the control group. The standard RR is expressed as:

$$Standard relative risk=\frac{Probability of cancer in case}{Probability of cancer in control}=\frac{\frac{Number of events in case}{Total number in case}}{\frac{Number of events in control}{Total number in control}}$$

RR equal to 1 suggests there is no difference in risk between the two groups. RR greater than one suggests there is a higher risk of the event occurring in the cancer group compared to the non-cancer group. RR less than 1 suggests there is a lower risk in the cancer group compared to the non-cancer group.

**Binomial relative risk**

The binomial RR is determined by dividing the risk in the specific subgroup by the risk in the general population. Binomial RR is defined as:

$$Binomial realtive risk=\frac{\frac{Number of events in case}{Number of events in case+Number of events in control}}{\frac{Total number in case}{Total number in case+Total number in control}}$$

This ratio indicates how much more (or less) likely cancer is to occur in the subgroup compared to the overall population. A binomial RR greater than one suggests that cancer is more likely in the subgroup than in the general population. A value less than 1 implies that cancer is less likely in the subgroup. A value of 1 indicates no difference in risk between the subgroup and the general population.

**Prevalence**

Prevalence is a measure of how widespread cancer is within a population at a given time. Prevalence is defined as:

$$Prevalence= \frac{Number of events in case}{Number of events in case+Number of events in control}$$

Prevalence provides necessary information about the burden of cancer on a population.

**Cohen’s effect size**

Cohen's d is a measure of effect size used to indicate the standardized difference between two means within subgroup populations (*e.g.*, the prevalence of females and males or White and Black patients). It is defined as:

$$\mathrm{Cohen}^{'}s d= \frac{\bar{X} - \bar{Y}}{S_{p}},$$

where $\bar{X}$ and $\bar{Y}$ are the means of subgroups and $S_{p}$is the pooled standard deviation of the two groups, calculated as the square root of the weighted average of the variances of the two groups. We categorized the effect size as follows: 0 to 0.33 represents a small effect, 0.34 to 0.66 represents a medium effect, and values greater than 0.67 indicate a large effect.

**Gender or race differences**

As a test statistic for assessing differences (whether based on gender, race, or area deprivation index (ADI)), we can measure the standard deviation (SD) of the difference between two log odds ratios (e.g., male and female, or Black and White individuals). This difference is expressed in units of pooled standard errors, which helps quantify the significance of observed disparities between the groups (Dervic E, 2021) :

$SD=\frac{log({OR}_{1})-log({OR}_{2})}{\sqrt{{SE}_{1}^{2}+{SE}_{2}^{2}}}$,

where SE represents the standard errors of the corresponding group. We evaluate the magnitude and statistical relevance of the differences across the specified groups. If |SD| > 5, the difference is considered highly significant, with P-values less than 0.00001.
