# Supplementary material for: Association between Diagnostic History and Cancer Incidence within 5 Years: A Real-world Observational Analysis
Source: Cancer Res Commun. 2026 May 11;6(5):1083–91. doi: 10.1158/2767-9764.CRC-26-0163 (PMC13158651; doi:10.1158/2767-9764.CRC-26-0163)
Supplement: Supplementary Table S1 — Table S1. Standard and binomial relative risks of ICD-10-CM medical diagnosis chapters across all patients. [file crc-26-0163_supplementary_table_s1_suppst1.docx]

Supplementary Appendix: Supplementary Table S1

**Table S1**. Standard and binomial relative risks of ICD-10-CM medical diagnosis chapters across all patients.

| **Chapter**  **(Code Range)** | **Description** | **Count** | | **Relative Risk** | |
| --- | --- | --- | --- | --- | --- |
|  |  | **Case** | **Control** | **Standard** | **Binomial** |
| 1 (A00-B99) | [Certain infectious and parasitic diseases](https://icd.codes/icd10cm/chapter1) | 87715 | 18185 | 1.08 | 1.01 |
| 3 (D50-D89) | [Diseases of the blood and blood-forming organs and certain disorders involving the immune mechanism](https://icd.codes/icd10cm/chapter3) | 5612 | 1117 | 1.13 | 1.02 |
| 4(E00-E89) | [Endocrine, nutritional and metabolic diseases](https://icd.codes/icd10cm/chapter4) | 170156 | 28308 | 1.35 | 1.05 |
| 5(F00-F99) | [Mental, Behavioral and Neurodevelopmental disorders](https://icd.codes/icd10cm/chapter5) | 296117 | 68003 | 0.97 | 0.99 |
| 6 (G00-G99) | [Diseases of the nervous system](https://icd.codes/icd10cm/chapter6) | 101477 | 23017 | 0.98 | 0.99 |
| 7 (H00-H59) | [Diseases of the eye and adnexa](https://icd.codes/icd10cm/chapter7) | 125852 | 31282 | 0.90 | 0.98 |
| 8 (H60-H95) | [Diseases of the ear and mastoid process](https://icd.codes/icd10cm/chapter8) | 119814 | 35719 | 0.75 | 0.94 |
| 9 (I00-I99) | [Diseases of the circulatory system](https://icd.codes/icd10cm/chapter9) | 80107 | 25589 | 0.70 | 0.93 |
| 10(J00-J99) | [Diseases of the respiratory system](https://icd.codes/icd10cm/chapter10) | 466672 | 95509 | 1.09 | 1.02 |
| 11 (K00-K95) | [Diseases of the digestive system](https://icd.codes/icd10cm/chapter11) | 166651 | 37024 | 1.00 | 1.00 |
| 12 (L00-L99) | [Diseases of the skin and subcutaneous tissue](https://icd.codes/icd10cm/chapter12) | 154845 | 28841 | 1.20 | 1.03 |
| 13 (M00-M99) | [Diseases of the musculoskeletal system and connective tissue](https://icd.codes/icd10cm/chapter13) | 542713 | 93600 | 1.30 | 1.04 |
| 14 (N00-N99) | [Diseases of the genitourinary system](https://icd.codes/icd10cm/chapter14) | 180588 | 24476 | 1.65 | 1.08 |
| 15 (O00-O9A) | [Pregnancy, childbirth and the puerperium](https://icd.codes/icd10cm/chapter15) | 108914 | 22030 | 1.11 | 1.02 |
| 16 (P00-P96) | [Certain conditions originating in the perinatal period](https://icd.codes/icd10cm/chapter16) | 56260 | 41532 | 0.30 | 0.70 |
| 17(Q00-Q99) | [Congenital malformations, deformations and chromosomal abnormalities](https://icd.codes/icd10cm/chapter17) | 66914 | 33884 | 0.44 | 0.81 |
| 18 (R00-R99) | [Symptoms, signs and abnormal clinical and laboratory findings, not elsewhere classified](https://icd.codes/icd10cm/chapter18) | 750098 | 183568 | 0.91 | 0.98 |
| 19 (S00-T88) | [Injury, poisoning and certain other consequences of external causes](https://icd.codes/icd10cm/chapter19) | 204031 | 57037 | 0.80 | 0.96 |
| 20(V00-Y99) | [External causes of morbidity](https://icd.codes/icd10cm/chapter20) | 553661 | 99691 | 1.24 | 1.04 |
| 21 (Z00-Z99) | [Factors influencing health status and contact with health services](https://icd.codes/icd10cm/chapter21) | 434842 | 97967 | 0.99 | 0.99 |
