# Supplementary material for: Association between Diagnostic History and Cancer Incidence within 5 Years: A Real-world Observational Analysis
Source: Cancer Res Commun. 2026 May 11;6(5):1083–91. doi: 10.1158/2767-9764.CRC-26-0163 (PMC13158651; doi:10.1158/2767-9764.CRC-26-0163)
Supplement: Supplementary Table S2 — Table S2. Top selected ICD-10-CM medical diagnoses across overall, gender, race, gender-race, and ADI patient groups. [file crc-26-0163_supplementary_table_s2_suppst2.docx]

Supplementary Appendix: Supplementary Table S2

**Table S2**. Top selected ICD-10-CM medical diagnoses across overall, gender, race, gender-race, and ADI patient groups. The most frequently appearing ICD-10-CM codes are N60 (benign mammary dysplasia), M34 (systemic sclerosis), and R43 (disturbances of smell and taste) for the overall data. In the stratified data, E78 (disorders of lipoprotein metabolism), N28 (kidney disorders), and R97 (abnormal tumor markers) are the most frequently appearing codes.

| **Cancer Type** | **Overall**  **data** | **Age >=40** | | | | | | | | | |
| --- | --- | --- | --- | --- | --- | --- | --- | --- | --- | --- | --- |
|  |  | **Gender** | | **Race** | | **Gender-race** | | | | **ADI** | |
|  |  | **Male** | **Female** | **Black** | **White** | **Male-**  **Black** | **Male**  **White** | **Female-Black** | **Female**  **White** | **Prosperity** | **Poverty** |
| **Overall**  **cancer** | N60  M34  R43 | R43  L57  I12 | N60 R43 M34 | N60  R43  Z67 | E78  R43  R97 | R43  L57  L82 | R97  E78  B19 | N60  Z67  R43 | L66  R39  N80 | E78  R43  R92 | E55  B20  E29 |
| **Breast** | I16  E78  M34 | - | R92  N64  E78 | R92  M35  N64 | N63  N64  E04 | - | - | R92  M67  M35 | N63  E04  N64 | N60  K85 | R67  E03 |
| **Skin** | S42  L57  E78 | L57  L67  N42 | S42  R92  B07 | M34  E87  M48 | O02  H40  020 | L57  D62  R47 | H40  N40  R10 | S42  R92  M94 | O02  O20  - | R97  R47 | N50  N40 |
| **Prostate** | R97  E78  R36 | R97  L82  M71 | - | R97  L28  L82 | R97  E78  N32 | L28  M71  F19 | R97  E78  J38 | - | - | R97  H16 | R93  N40 |
| **Lung** | M34  H02  L97 | M12  M75  R97 | H02  I21  N18 | H02  I21  R97 | K74  I71  N18 | M94  M12  H26 | I71  M75  K74 | H02  H80  L90 | N18  J44  F17 | Z76  H02 | H00  M25 |
| **Colorectal** | L97  I21  E78 | F03  J45  E03 | R32  N18  L97 | L97  K46  N18 | F03  G47  R50 | J45  E03  J32 | R50  G47  - | N18  R32  L97 | H04  M79  = | N13  F03 | I73 |
| **Liver** | Q44  I85  K70 | I37  K74  R93 | I85  K74  K76 | I37  K74  N41 | K74  K76  - | N32  J47  K74 | K74  H40 | I84  K74  K72 | K74  K76  D86 | K74  K70 | K74  M25 |
| **Bladder** | M48  N28  E78 | T84  N28  J33 | M48  L40  I73 | N28  R84  L40 | M21  B35  - | N28  T84  J33 | M12  B35  E29 | K80  I73  R76 | R31  M76  L84 | K12  N28 | J40  I70 |
| **Diffuse NHL** | H34  K58  E10 | E10  M35  K22 | I20  K57  B02 | E10  B02  I20 | - | J32  R10  E78 | - | I20  B02  K57 | O26  N89  J32 | N89  I70 | K44 |
| **Leukemia** | L93  M48  E70 | M17  I20  H40 | L40  M48  I73 | L40  M35  I73 | - | M17  H40  I20 | - | L40  F70  M67 | J45  H40  N18 | J01  T50 | H40 |
| **Kidney** | N28  H17  M12 | H17  N28  H35 | N28  R53  H90 | H17  N28  H40 | - | H17  N28  M50 | B35 | N28  M24  M19 | N28  L68  L70 | N28  M12 | H40  J40 |
| **Thyroid** | A31  E21  E04 | E04  F25  E11 | E05  G20  R91 | A31  E05  E04 | G20  F25  - | E04  E11  R35 | - | M47  F07  J41 | N83  R13  R49 | H35  E04 | F33 |
| **Follicular NHL** | H34  H35  I20 | E10  M25  R10 | I20  J01  K57 | E10  I20  K57 | N20  O26  N89 | R10  E78  H52 | - | I10  K57  R35 | O26  N89 | N87  I70 | K44 |
| **Pancreatic** | M48  K86  G35 | K86  K64  M47 | E78  F41  F43 | K86  R31  K21 | M48  G35  F41 | K85  K64  K21 | N51  M47 | M77  R31  E78 | M25  M79  M17 | K82  G24 | G60  M17 |
| **Endometrial** | F22  N95  E78 | L97 | F22  G47  N95 | K34  N95  G47 |  | - | - | B30  K35  N89 | F22  N95 | G47  B30 | - |
| **Ovarian** | R97  M50  N83 |  | M78  M77  F25 | M76  M77  H52 | F35 | - | - | M76  M77  E04 | F43  F25  J06 | F32  F41  N83 | O26 |
| **Stomach** | F44  R49  I21 | F46  M95  M51 | F44  R49  J38 | F44  J38  R49 | J33  K92 | - | - | F44  J38  R49 | -  - | J33  A09 | M51 |
| **Tongue** | M10  E78 | M10 | E46  N94  H92 | M25 | M65  M10  M19 | E78  L03 | M10 | H92  R69  J01 | - | E78  M17 | R10 |
| **Tonsil** | M79 | M79 | H59  H91 | M75  M25 |  | M79 | - | H69  H91 | J44 | M25  M79 | - |
| **Anal** | K50  K62  M72 | M12  Q66  M25 | N28  F33  R03 | M25  N28 | Q66  G57  M19 | - | - | - | - | M25  M77 | - |
| **Oropharyngeal** | E01 | F11 | - | K43 | E01 | - | - | - | - | N28 | - |
