# Supplementary material for: Association between Diagnostic History and Cancer Incidence within 5 Years: A Real-world Observational Analysis
Source: Cancer Res Commun. 2026 May 11;6(5):1083–91. doi: 10.1158/2767-9764.CRC-26-0163 (PMC13158651; doi:10.1158/2767-9764.CRC-26-0163)
Supplement: Supplementary Figure S1 — Figure S1. CONSORT diagram summarizing the data inclusion and exclusion process. [file crc-26-0163_supplementary_figure_s1_suppsf1.docx]

Supplementary Appendix: Supplementary Figure S1


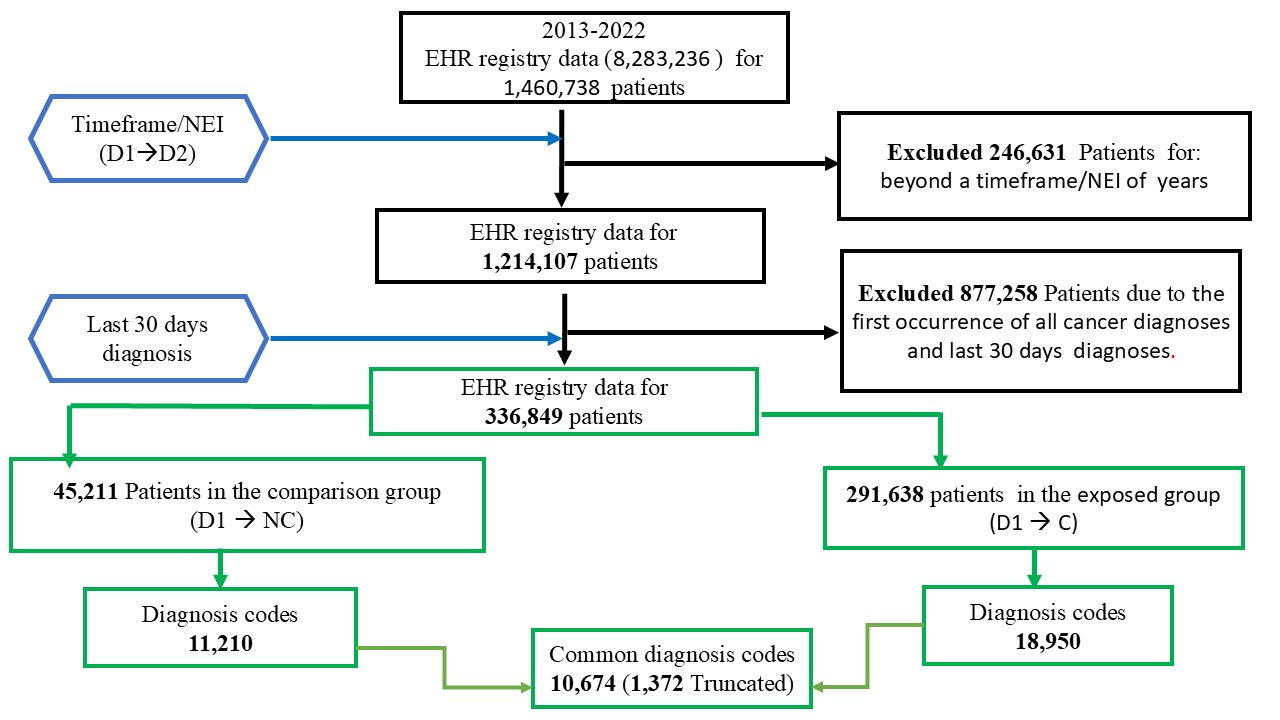


**Figure S1.** CONSORT diagram summarizing the data inclusion and exclusion process.
