# Supplementary material for: Association between Diagnostic History and Cancer Incidence within 5 Years: A Real-world Observational Analysis
Source: Cancer Res Commun. 2026 May 11;6(5):1083–91. doi: 10.1158/2767-9764.CRC-26-0163 (PMC13158651; doi:10.1158/2767-9764.CRC-26-0163)
Supplement: Supplementary Figure S3 — Figure S3. A clustered heatmap of 55 codes, each with at least one RR greater than 2.0 (with a significant confidence interval) across three age groups. [file crc-26-0163_supplementary_figure_s3_suppsf3.docx]

Supplementary Appendix: Supplementary Figure S3


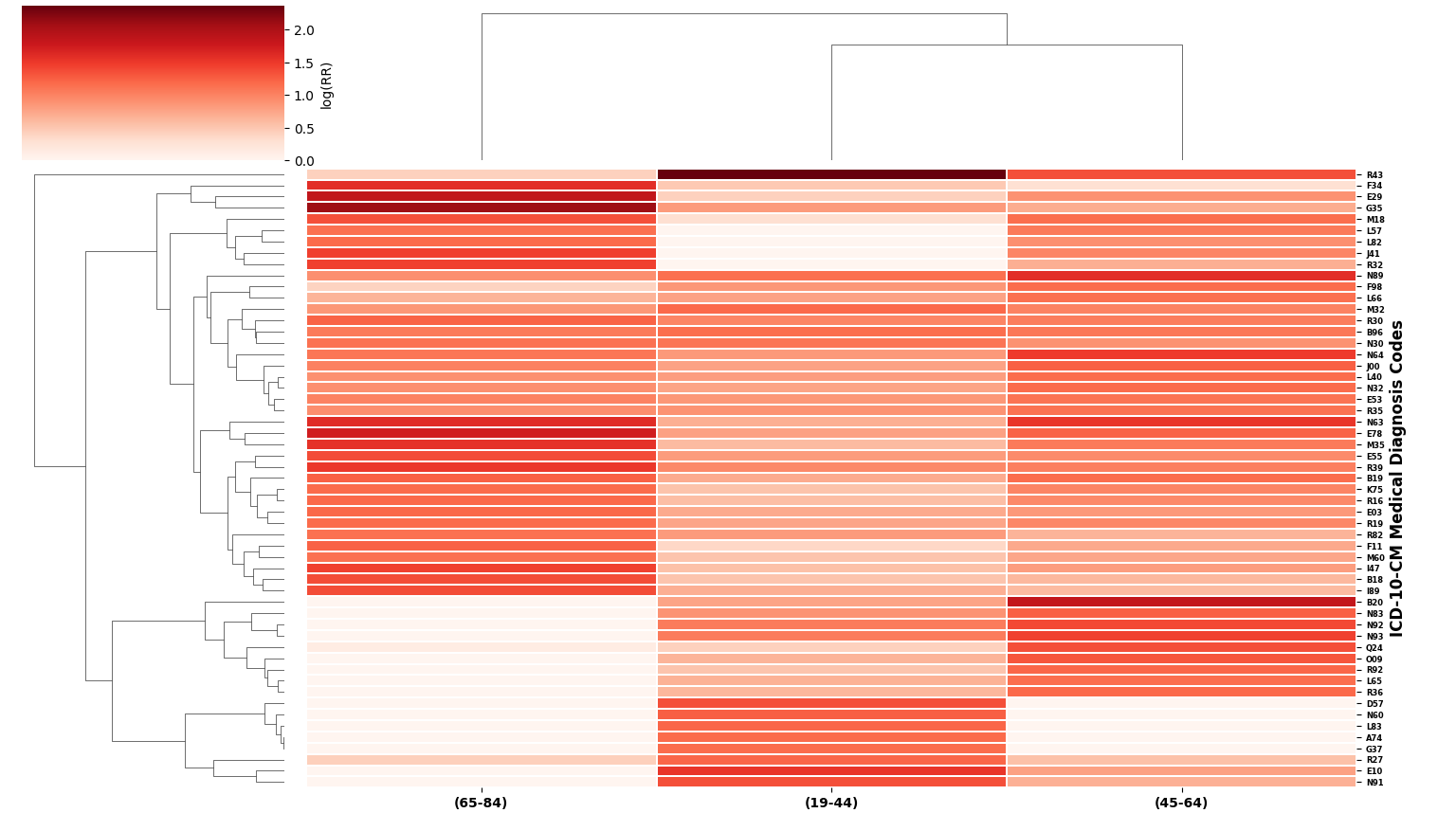


**Figure S3.** A clustered heatmap of 55 codes, each with at least one RR greater than 2.0 (with a significant confidence interval) across three age groups.
