# Supplementary material for: Association between Diagnostic History and Cancer Incidence within 5 Years: A Real-world Observational Analysis
Source: Cancer Res Commun. 2026 May 11;6(5):1083–91. doi: 10.1158/2767-9764.CRC-26-0163 (PMC13158651; doi:10.1158/2767-9764.CRC-26-0163)
Supplement: Supplementary Figure S4 — Figure S4. A hierarchical clustering heatmap of 98 ICD-10-CM codes (RR >2.0) across four demographic groups. [file crc-26-0163_supplementary_figure_s4_suppsf4.docx]

Supplementary Appendix: Supplementary Figure S4


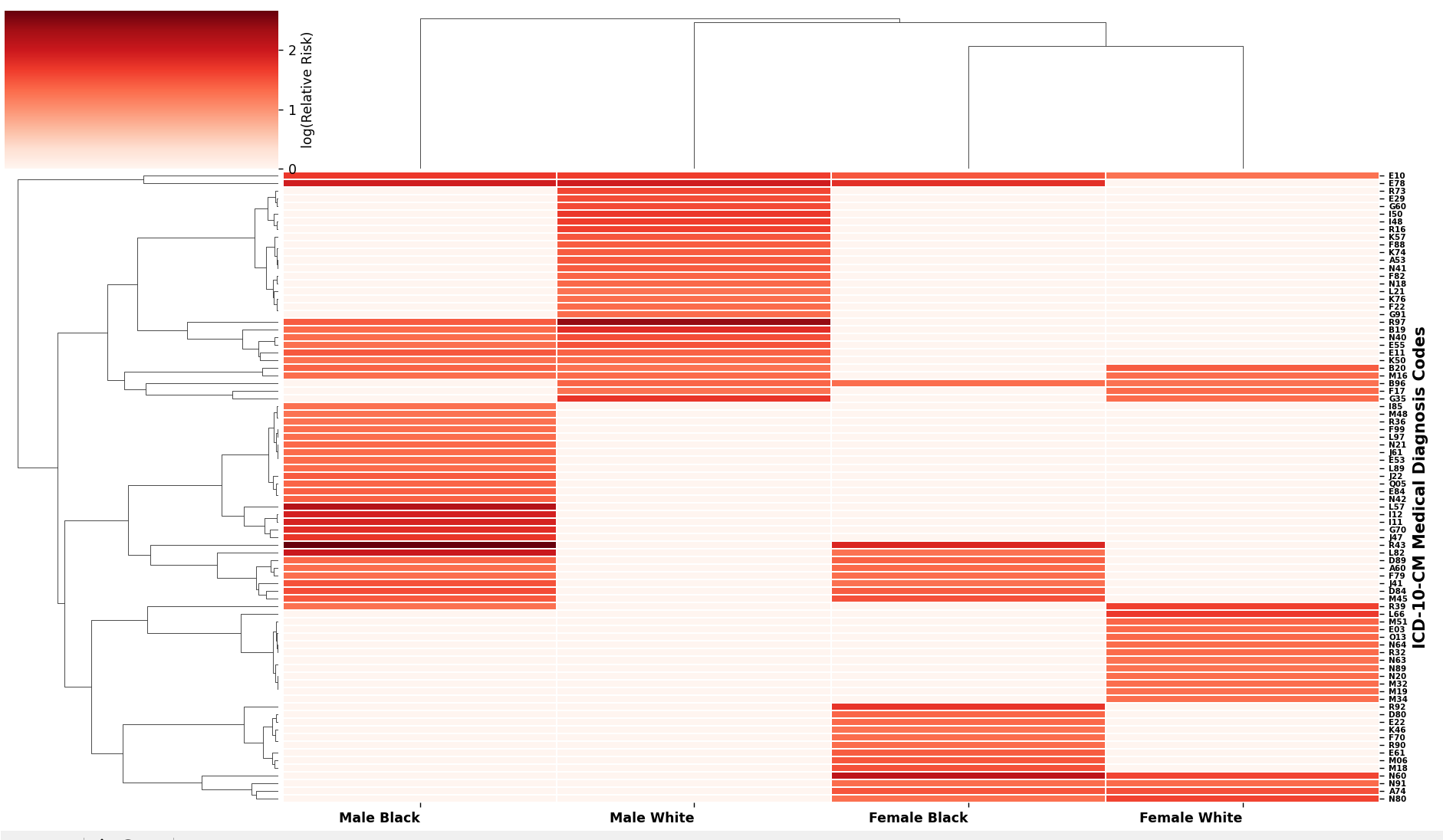


**Figure S4.** A hierarchical clustering heatmap of 98 ICD-10-CM codes (RR >2.0) across four demographic groups. Type 1 diabetes (E10) is the only HC strongly linked across all groups, while lipoprotein metabolism disorders (E78) are common in three. Males in both racial groups show strong associations with seven HCs, including abnormal tumor markers (R97), viral hepatitis (B19), and benign prostatic hyperplasia (N40).
