# Supplementary material for: Association between Diagnostic History and Cancer Incidence within 5 Years: A Real-world Observational Analysis
Source: Cancer Res Commun. 2026 May 11;6(5):1083–91. doi: 10.1158/2767-9764.CRC-26-0163 (PMC13158651; doi:10.1158/2767-9764.CRC-26-0163)
Supplement: Supplementary Figure S5 — Figure S5. Radial plot comparing relative risks (RR) for 20 cancer types between low-ADI and high-ADI populations. [file crc-26-0163_supplementary_figure_s5_suppsf5.docx]

Supplementary Appendix: Supplementary Figure S5


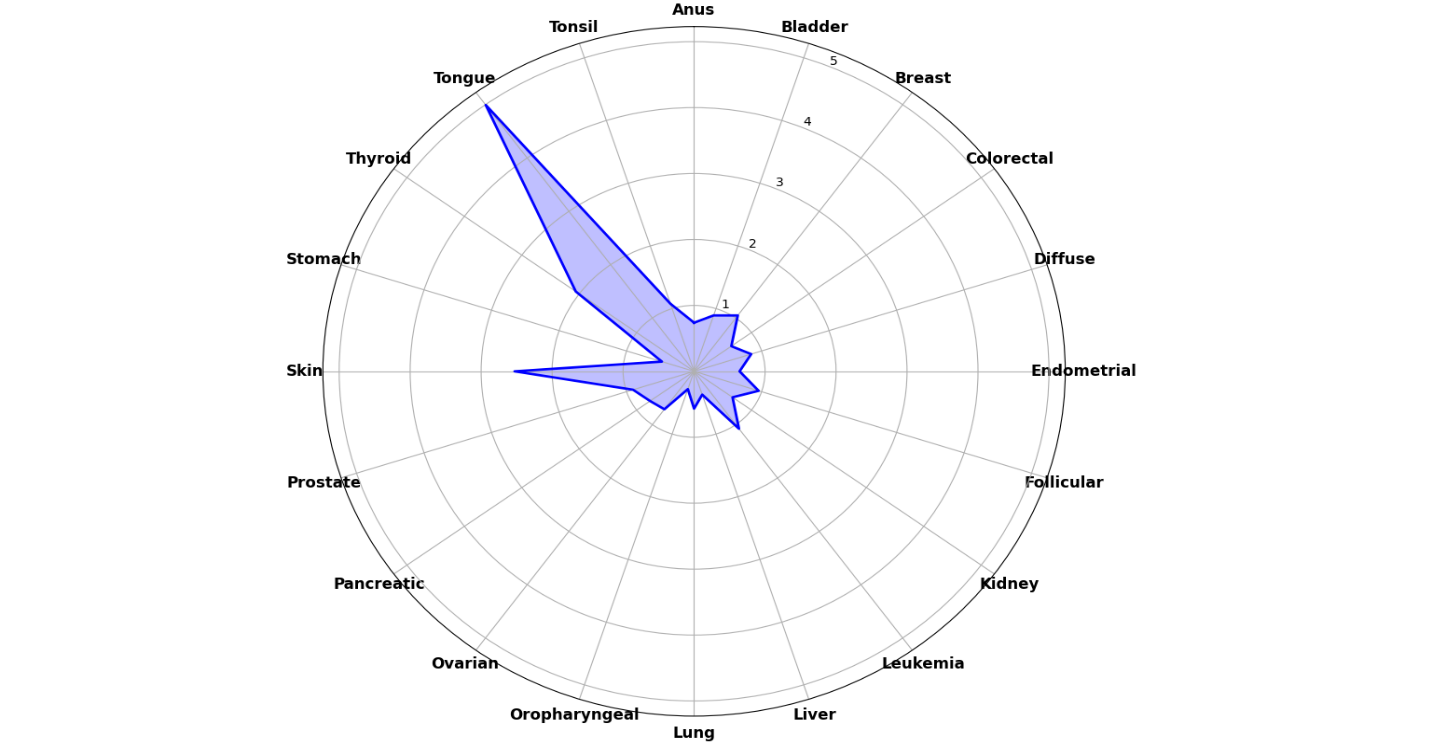


**Figure S5.** Radial plot comparing relative risks (RR) for 20 cancer types between low-ADI and high-ADI populations. The plot shows that individuals residing in high-ADI areas exhibit substantially higher RR for thyroid and skin cancers compared with those in low-ADI areas, while most other cancer types show modest or minimal ADI-related differences.
