# Supplementary material for: Association between Diagnostic History and Cancer Incidence within 5 Years: A Real-world Observational Analysis
Source: Cancer Res Commun. 2026 May 11;6(5):1083–91. doi: 10.1158/2767-9764.CRC-26-0163 (PMC13158651; doi:10.1158/2767-9764.CRC-26-0163)
Supplement: Supplementary Figure S6 — Figure S6. A clustered heatmap of socioeconomic factors' impact on cancer diagnoses in low and high ADI. [file crc-26-0163_supplementary_figure_s6_suppsf6.docx]

Supplementary Appendix: Supplementary Figure S6


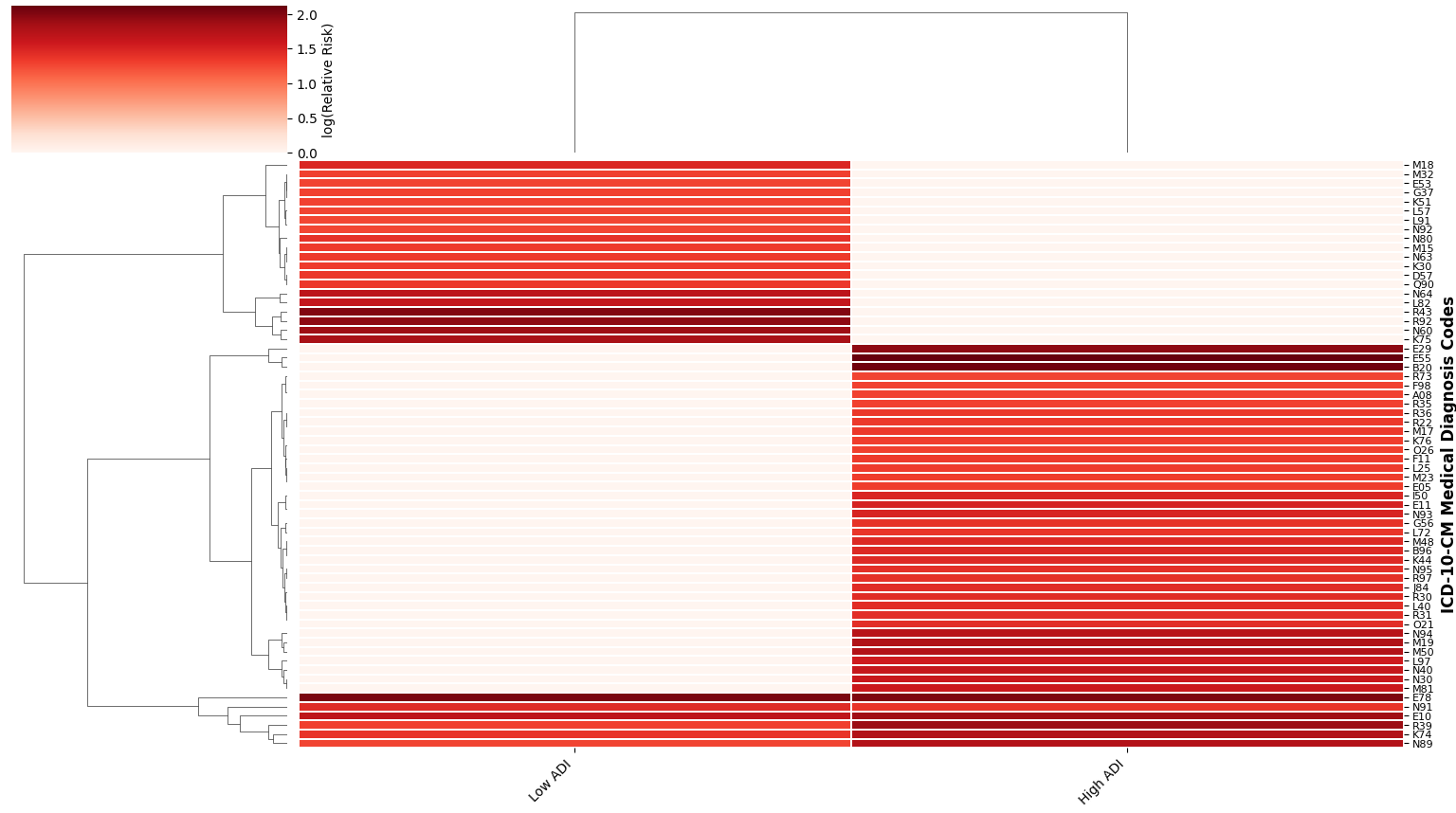


**Figure S6:** A clustered heatmap of socioeconomic factors' impact on cancer diagnoses in low and high ADI (RR >2.0). Six conditions, including lipoprotein metabolism disorders (E78) and Type 1 diabetes (E10), show high cancer risk in both groups. Eighteen conditions, such as disturbances of smell/taste (R43) and benign mammary dysplasia (N60), are linked to low ADI, while 36, including testicular dysfunction (E29) and HIV disease (B20), are linked to high ADI.
