# Supplementary material for: Association between Diagnostic History and Cancer Incidence within 5 Years: A Real-world Observational Analysis
Source: Cancer Res Commun. 2026 May 11;6(5):1083–91. doi: 10.1158/2767-9764.CRC-26-0163 (PMC13158651; doi:10.1158/2767-9764.CRC-26-0163)
Supplement: Supplementary Figure S7 — Figure S7. Graphical user interface (GUI) of the rule-based artificial intelligence system developed for 5-year cancer susceptibility estimation. [file crc-26-0163_supplementary_figure_s7_suppsf7.docx]

Supplementary Appendix: Supplementary Figure S7


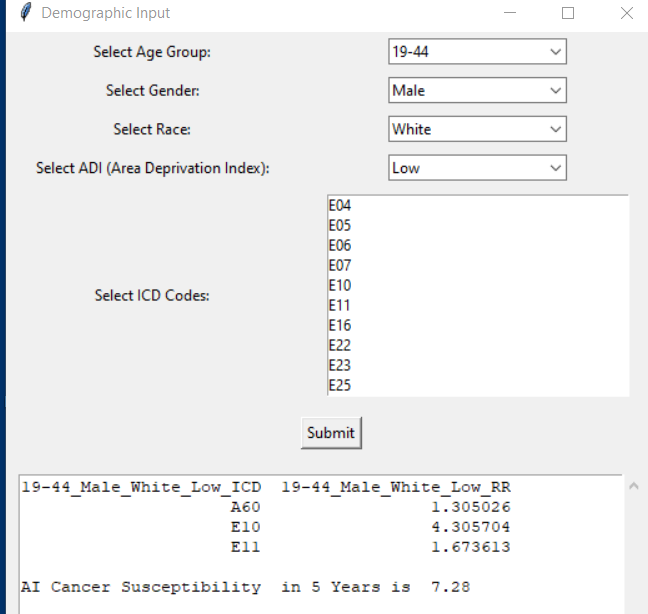


Figure S7. Graphical user interface (GUI) of the rule-based artificial intelligence system developed for 5-year cancer susceptibility estimation. The interface allows users to input demographic characteristics (age group, gender, race), socioeconomic context (Area Deprivation Index, ADI), and preexisting health conditions (ICD codes). Upon submission, the rule engine evaluates predefined IF–THEN logic rules derived from epidemiologic associations and computes relative risk (RR) contributions for each triggered condition. The final cancer susceptibility score is generated through deterministic rule aggregation without any machine learning training procedure. The system provides full transparency by displaying individual ICD-specific risk contributions and the overall 5-year susceptibility estimate. These findings should be interpreted carefully, as ADI may not fully capture individual-level socioeconomic or environmental exposures, and the lack of tobacco data may introduce residual confounding.
